# Somatic Arc protein expression in hippocampal granule cells is increased in response to environmental change but independent of task-specific learning

**DOI:** 10.1038/s41598-017-12583-1

**Published:** 2017-09-29

**Authors:** J. P. Cleland, E. F. Willis, P. F. Bartlett, J. Vukovic

**Affiliations:** 1 School of Biomedical Sciences, The University of Queensland, Brisbane, Queensland 4072 Australia; 2 The Queensland Brain Institute, The University of Queensland, Brisbane, Queensland 4072 Australia

## Abstract

Activated neurons express immediate-early genes, such as Arc. Expression of Arc in the hippocampal granule cell layer, an area crucial for spatial learning and memory, is increased during acquisition of spatial learning; however, it is unclear whether this effect is related to the task-specific learning process or to nonspecific aspects of the testing procedure (e.g. exposure to the testing apparatus and exploration of the environment). Herein, we show that Arc-positive cells numbers are increased to the same extent in the granule cell layer after both acquisition of a single spatial learning event in the active place avoidance task and exploration of the testing environment, as compared to naïve (i.e. caged) mice. Repeated exposure the testing apparatus and environment did not reduce Arc expression. Furthermore, Arc expression did not correlate with performance in both adult and aged animals, suggesting that exploration of the testing environment, rather than the specific acquisition of the active place avoidance task, induces Arc expression in the dentate granule cell layer. These findings thus suggest that Arc is an experience-induced immediate-early gene.

## Introduction

Immediate-early genes (IEGs) are rapidly and transiently upregulated in neurons activated by physiological and supraphysiological stimuli, such as behavioral experience or high-frequency stimulation^1–3^. The protein products of IEGs are divided into two classes: transcription factors that regulate transcription of target genes, and effectors which directly regulate a range of cellular functions. A growing body of evidence indicates these proteins make important contributions to the cellular and molecular mechanisms that underpin learning and memory, in addition to serving as robust markers of recent neuronal activity^4^. In particular, the effector Arc (also known as Arg3.1) has been demonstrated to be critical for the consolidation of new memories^1,5–10^.

Every aspect of Arc expression, from transcription to protein degradation, is tightly regulated by a complex array of signaling cascades. Following activity-dependent signaling through the N-methyl-D-aspartate (NMDA) receptor, Arc mRNA is significantly upregulated in the nucleus before being transported to the dendrites for translation^1^. Arc protein is highly enriched at the postsynaptic density, where it has a number of important functions in synaptic plasticity, including regulation of α-amino-3-hydroxy-5-methyl-4-isoxazolepropionic acid (AMPA) receptor trafficking^11^. Consistent with this, Arc knockout mice or rats infused with Arc antisense oligodeoxynucleotides exhibit impaired late-phase long-term potentiation (LTP) and spatial memory consolidation^6,12^. These properties have led to the emergence of Arc as a marker of neuronal activity and synaptic plasticity during specific behaviors, such as acquisition or retrieval of a spatial task, in which a goal must be achieved using spatial information^3,6,12–15^.

To date, the majority of studies on Arc expression and behavior have focused on exploration of a novel environment: typically, an open arena within a square box. These studies demonstrated that exploration of a novel environment resulted in upregulated Arc expression throughout the hippocampus, which was maintained for several hours^2,7,15^. Although exploration of a novel environment may be considered a type of spatial learning, challenging alternatives, such as acquisition of spatial learning, undoubtedly provide more functionally meaningful information^15–19^.

Our laboratory previously demonstrated that genetic ablation of doublecortin-expressing immature dentate granule cells is associated with a deficit in the acquisition of the active place avoidance (APA) spatial learning task, and a downregulation of somatic Arc expression in the dentate granule cell layer^20^. Although further studies are required to precisely delineate the relationship between adult neurogenesis and Arc expression, our previously published work demonstrates Arc-expressing (Arc^+^) hippocampal neurons may be important for spatial learning^20^. The current prevailing model is that immature granule cells, which are more excitable and more amenable to synaptic plasticity than their mature counterparts, influence the activity (and thus IEG expression) of the mature granule cells by differentially modulating inhibitory interneurons and excitatory mossy cells^21–25^. Consistent with this possibility, Guzowski and colleagues reported that after acquisition of the Morris water maze task, Arc mRNA levels in the whole dorsal hippocampus positively correlated with performance in the task; however, a correlation between task performance and Arc expression in specific hippocampal subregions could not be determined due to limited spatial resolution^3^. Arc expression has also been demonstrated to be important for hippocampal-dependent memory for fear conditioning^26,27^. Indeed, using optogenetics Denny and colleagues demonstrated that silencing Arc^+^ hippocampal neurons during contextual fear conditioning blocked subsequent fear memory recall, indicating that the Arc^+^ subpopulation is also important for this behavior^9^.

Although it is without question that Arc expression relates to behavioral experiences, there is presently little evidence addressing the question of whether there are actually learning-specific changes in Arc expression in the hippocampus following acquisition of spatial learning. Guzowski and colleagues demonstrated that Arc expression is upregulated in the dentate granule cell layer, CA1 and CA3 after acquisition of the Morris water maze task^3^; however, the use of ‘caged’ controls (mice sacrificed immediately after removal from their home cage) prevented the authors from determining whether the change in Arc expression was specifically related to learning. Several questions thus remain unanswered: Is Arc expression selectively upregulated in the hippocampus following acquisition of spatial learning? And, if so, is such an effect localized to a specific hippocampal cell subpopulation? The study presented herein aimed to answer these questions; to do so, we utilized the APA task with a novel control condition; specifically, mice could freely explore the testing environment with the shock zone turned off (i.e. ‘exposure only control group’ = Exp Ctrl), which addresses many of the aspects of behavioral testing unable to be tested under caged conditions. We report that Arc^+^ cell numbers within the dentate gyrus are not different after APA acquisition compared to exposure only control group, and also that the number of prior exposures had no impact on somatic Arc protein expression. Furthermore, Arc^+^ granular cells are upregulated shortly after introduction to a new environment, suggesting that somatic Arc expression may be a generalized experience-induced marker of deprivation, rather than a specific reporter of goal-directed spatial learning. These findings address several gaps in our understanding as to how induction of somatic Arc expression in hippocampal neurons relates to the acquisition of spatial learning as opposed to exploration. In addition, we have examined the spatial learning performance of aged 25-month-old animals, and its relationship to somatic Arc expression.

## Materials and Methods

### Animals

Male C57BL/6 J mice aged either 10–15 weeks (‘young adult’) or 25-months-old (‘aged’) were used for all experiments. Mice were housed individually and maintained on a 12-h light/dark cycle with food and water provided *ad libitum*. All experiments were conducted in accordance with the Australian Code for the Care and Use of Animals for Scientific Purposes, with approval from The University of Queensland’s Animal Ethics Committee.

### Behavioral apparatus and procedure

Before behavioral testing, to minimize handling-related stress and anxiety, mice were handled daily, for 30 s to 1 min, over seven consecutive days. Hippocampal-dependent spatial learning was assessed using the APA task, as described previously^20^. The APA apparatus (Bio-Signal Group) consists of an elevated arena, 77-cm in diameter, with a grid floor fenced by a 32 cm-high transparent circular boundary. The arena rotates counter-clockwise (one rotation per minute) and a 60° shock zone remains constant within the arena (i.e. does not rotate). On the walls of the room in which the arena is housed are visual cues (large black and white symbols/shapes); using these cues, the mouse must determine the position of the shock zone relative to its location. Entrance into the shock zone triggers the delivery of a brief foot shock (500 ms, 60 Hz, 0.5 mA). All mice leave the shock zone immediately following administration of the foot shock; no freezing was observed. In the present study, the position of the mouse was tracked using an overhead camera and Tracker software (Bio-Signal Group; version 2.36). To limit odor cues as a potential factor influencing performance in the APA task, the metal grid, underlying floor, and walls of the testing arena were cleaned with 70% ethanol before each trial.

Mice were habituated to the testing environment by being allowed to explore the rotating arena, with the shock zone turned off, for 20 min. Approximately 24 h later, mice were either tested in the APA task for 20 min (‘APA’ mice), or habituated for an additional 20 min (‘Exp Ctrl’ mice). Following testing, mice were returned to their home cage. ‘Caged’ control mice were handled for seven consecutive days. Twenty-four hours after the final handling session, brains were collected for immunofluorescence analyses, as described below. To test whether experiencing more subtle differences in the environment, e.g. a cage change, also impacts on somatic Arc expression, a separate cohort of so-called ‘new cage’ mice were transferred from their home cage to a new clean cage for either 20 min or 16 h. Following this, mice were sacrificed as detailed below and their brains collected for immunofluorescence analyses. Neither caged nor new cage mice underwent behavioral testing.

### BrdU-labeling of adult-born neurons

To label adult-born neurons, mice were injected intraperitoneally with 5-bromo-2’-deoxyuridine (BrdU; Sigma; 100 mg/kg body weight) dissolved in 1.75 mM sodium hydroxide (NaOH) solution twice daily (6 h apart), for 5 consecutive days^28–30^. Mice were perfused 1, 2, or 4 weeks after the last BrdU injection following behavioral testing to label 0–1 week old, 1–2 week old, or 4–5 week old cells, respectively (Fig. 4A). For BrdU staining, sections underwent a DNA denaturation step involving incubation in pre-heated 1 M hydrochloric acid for 45 min at 45 °C, 10 min wash in 0.1 M boric acid and PBS, followed by incubation in blocking solution, as described below.

### Tissue processing and analysis

Unless specified otherwise, mice were deeply anesthetized at 90 min post-experience via intraperitoneal injection of sodium pentobarbitone (150 mg/kg; Virbac), before being transcardially perfused with 20 mL of 0.1 M phosphate buffered saline (PBS; pH 7.4), followed by 30 mL of 4% paraformaldehyde (PFA; pH 7.4)^31^. Brains were post fixed in 4% PFA (pH 7.4) at 4 °C for 24 h, before being cryoprotected in 30% sucrose solution and serially sectioned in a coronal orientation at a thickness of 40 µm using a sliding microtome (Leica).

Immunofluorescence was performed on free-floating sections (1 in 6 series). In brief, sections were incubated for 1 h in a blocking solution (5% normal goat serum, 0.3% Triton X-100 in PBS), then incubated overnight at 4 °C in primary antibodies (guinea pig anti-Arc, 1:1000, Synaptic Systems; mouse anti-BrdU, 1:500, Roche) diluted in PBS with 0.3% Triton X-100. Sections were then washed in PBS and incubated for 2 h in secondary antibodies (goat anti-mouse Alexa Fluor 647, 1:1000, Life Technologies; goat anti-guinea pig biotinylated IgG, 1:1000, Vector Laboratories) diluted in PBS with 0.3% Triton X-100 and the nuclear stain DAPI (1:5000, Sigma), followed by three washes in PBS and incubation in Streptavidin-Cy3 (1:1000; Sigma). Sections were washed in PBS, before being mounted in fluorescence mounting medium (Dako) and cover slipped.

Arc^+^ cells in the dentate granule cell layer were imaged using an Axio Imager Z2 microscope with an ApoTome structured illumination attachment (Zeiss) and an AxioCam MRm camera (Zeiss). Z-stacks (21 µm total range) of seven to eight sections per brain were acquired at 20x magnification, with Arc^+^ cells quantified using maximum intensity projections generated using Zen Blue (Zeiss). Live counting of BrdU-positive (BrdU^+^) and BrdU^+^/Arc^+^ cells in the dentate granule cell layer was conducted using an Axio Imager Z2 microscope (Zeiss) and an ORCA-R2 digital charge-coupled device camera (Hamamatsu) with StereoInvestigator (MicroBrightfield Bioscience) software. BrdU^+^ and BrdU^+^/Arc^+^ cells were identified at 40x magnification, with cells considered to be positive if somatic (Arc) or nuclear (BrdU) signal stood out clearly from surrounding tissue. Cell counts were normalized to the dentate granule cell layer length. Sections along the rostrocaudal axis of the dentate granule cell layer were selected according to their position relative to the anatomical landmark bregma (rostral dentate granule cell layer: 1.34–2.46 mm; caudal dentate granule cell layer: 2.54–3.52 mm)^32^. Arc^+^ granule cells were quantified in the following domains of the dentate granule cell layer: total upper, total lower, total rostral, and total caudal blade; as well as in the following subdomains: upper rostral, upper caudal, lower rostral, and lower rostral blade. All counting was performed by investigators blinded to the experimental conditions.

### Data analysis

All statistical analyses were performed using GraphPad Prism software (version 7.02). For comparisons between three or more groups, non-repeated or repeated one-way or two-way analyses of variance (ANOVA) with Tukey’s or Bonferroni’s *post hoc* tests were used as specified. For repeated measures ANOVA, we used the Greenhouse-Geisser correction. For comparisons between two groups, unpaired two-tailed t-tests with Welch’s correction were used. To examine the putative relationship between somatic Arc expression and APA performance, linear regressions were conducted and the Pearson’s R^2^ coefficient reported. Statistical significance was set at *P* < 0.05. All data are represented as the mean ± the standard error of the mean (SEM), and individual animals are shown as dot points.

## Results

### Arc expression is upregulated in the dentate granule cell layer following both acquisition of the APA task and exploration of the testing environment

**Figure 1:**
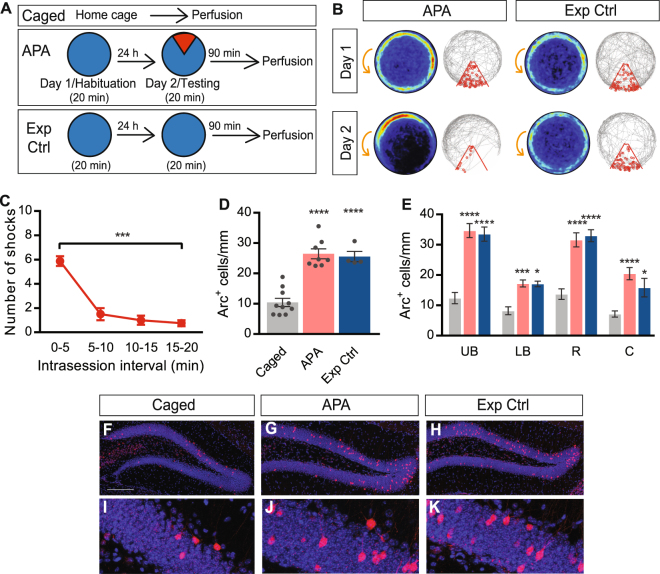
Number of Arc^+^ cells in the granule cell layer is increased independent of task-specific learning. **(A)** Schematic representation of the experimental approach. Arena is indicated in blue, while the shock zone is indicated in red. **(B)** Heat maps and representative tracks during Day 1 and Day 2 of APA (left) and Exp Ctrl (exposure control, right) mice, showing APA mice developing place-specific avoidance for the shock zone. Heat maps represent the merged maps of all mice within each experimental group, where the color of a pixel represents the average trajectory of tracks at that location (blue = low, red = high proportion of time). Gray line indicates a path of a single representative mouse and each entry into the shock zone (red sector) is visualized by a small red circle. Orange arrow indicates the counter-clockwise rotation of the testing arena. **(C)** Performance in the APA task. **(D)** Quantification of somatic Arc protein expression in the granule cell layer of caged, APA, and Exp Ctrl mice. **(E)** Quantification of Arc protein expression in different subdomains of the granule cell layer of caged (grey bars), APA (red bars), and Exp Ctrl (blue bars) mice. All comparisons are between caged and APA or Exp Ctrl groups. **(F–K)** Representative images of Arc^+^ cells (red) in the dentate granule cell layer of caged **(F)**, APA **(G)**, and Exp Ctrl **(H)** mice. All cells are labeled with the nuclear stain DAPI (shown in blue). **(I–K)** Enlarged views of boxed regions in **F–H**, respectively. Data represent mean ± SEM. Scale bars represent 200 µm. *P < 0.05, ***P < 0.001, ****P < 0.0001. UB, upper blade; LB, lower blade; R, rostral; C, caudal.

To examine whether there are learning-specific changes in Arc expression in the dentate granule cell layer after acquisition of the APA task, mice were tested either with the shock zone turned on (‘APA’ mice) or off (‘Exp Ctrl’ mice). A schematic overview of the experimental design is shown in Fig. 1A. APA mice quickly learned to avoid the shock zone over the course of a single 20-min session, as evidenced by a significant decrease in the number of shocks received in the last 5 min, compared with the first 5 min, of the test (repeated measures one-way ANOVA, *F*_2.05,14.34_ = 35.46; *P* < 0.0001, Bonferroni’s *post hoc* analysis: *P* = 0.0003 for 0–5 min: 5.88 ± 0.40 shocks vs. 15–20 min: 0.75 ± 0.25 shocks; n = 8; Fig. 1B,C). Following this single learning event, there was a significant 155% increase in Arc^+^ granule cell numbers in APA mice compared with caged controls (one-way ANOVA, *F*_2,19_ = 36.81; *P* < 0.0001; Tukey’s *post hoc* analysis: *P* < 0.0001 for APA: 26.49 ± 1.64 cells/mm (n = 8) vs. caged: 10.41 ± 1.36 cells/mm (n = 10)). There was also a significant increase in Arc^+^ cells in the Exp Ctrl group compared with caged controls (~146% increase; one-way ANOVA: *F*_2,19_ = 36.81; *P* < 0.0001; Tukey’s *post hoc* analysis: *P* < 0.0001 for Exp Ctrl: 25.56 ± 1.70 cells/mm (n = 4) vs caged: 10.41 ± 1.36 cells/mm (n = 10); Fig. 1D,F–K). There was no correlation between number of Arc^+^ granule cells and the distance the mice travelled during the test (R^2^ = 0.13, *P* = 0.73).

To determine whether there were subtle, region-specific differences in Arc expression between experimental groups, the number of Arc^+^ granule cells in the total upper blade, total lower blade, total rostral, and total caudal domains of the dentate granule cell layer were compared. Arc expression was upregulated in all dentate granule cell layer subdomains of both APA and Exp Ctrl mice compared to caged controls (repeated measures two-way ANOVA: *F*_2,19_ = 34.51, *P* < 0.0001 for treatment; see Fig. 1E; however, there was no difference in the number of Arc^+^ cells within the dentate subdomains between APA mice compared with Exp Ctrl mice (repeated measures two-way ANOVA: Bonferroni’s *post hoc* analysis: upper blade *P* > 0.99, lower blade *P* > 0.99, rostral *P* > 0.99, caudal *P* = 0.33, APA (n = 8) vs. Exp Ctrl (n = 4), Fig. 1E). When comparing differences between subdomains, there were significantly more Arc^+^ granule cells in the upper blade than the lower blade (repeated measures two-way ANOVA, *F*_2,57_ = 107.4, *P* < 0.0001 for brain region, Bonferroni’s *post hoc* analysis: caged *P* = 0.014, APA *P* < 0.0001, Exp Ctrl *P* < 0.0001)^33^ and there were significantly more Arc^+^ granule cells in the rostral than the caudal granule cell layer (Bonferroni’s *post hoc* analysis: caged *P* < 0.0001, APA *P* < 0.0001, Exp Ctrl *P* < 0.0001). Additionally, there was no correlation between APA performance and Arc^+^ numbers in the dentate granule cell layer (total number of shocks received in a session vs. number of Arc^+^ cells: *P* = 0.37, R^2^ = 0.14; number of shocks received in the 15–20-min interval vs. number of Arc + cells: *P* = 0.86, R^2^ = 0.0059). Taken together, these data suggest Arc expression is upregulated in the dentate granule cell layer not only following acquisition of the APA task, but also following exploration of the testing environment.

### Upregulation of Arc protein expression in the dentate granule cell layer is not altered by repeat exposure to the testing environment

**Figure 2:**
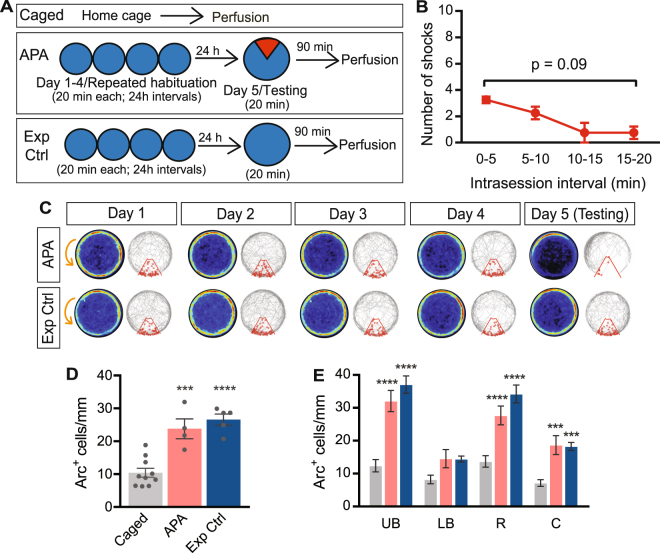
Five exposures to the experimental arena is insufficient to reduce the number of Arc^+^ granule cells. **(A)** Schematic representation of the experimental approach. Arena is indicated in blue, while the shock zone is indicated in red. **(B)** Performance in the APA task following repeated exposure to the testing arena. **(c)** Heat maps and representative tracks during the 4 days of repeated exploration of the arena and the test (day 5) of APA (top) and Exp Ctrl (exposure control, bottom) mice, showing APA mice developing place-specific avoidance for the shock zone on the test. Heat maps represent the merged maps of all mice within each experimental group, where the color of a pixel represents the average trajectory of tracks at that location (blue = low, red = high proportion of time). The gray line represents the path of a single representative mouse and each entry into the shock zone (red sector) is visualized by a small red circle. Orange arrow indicates the counter-clockwise rotation of the testing arena. **(D)** Quantification of overall Arc expression in the granule cell layer of caged, APA and Exp Ctrl mice, after repeated habituation. **(E)** Quantification of Arc protein expression in different subdomains of the granule cell layer of caged (grey bars), APA (red bars), and Exp Ctrl (blue bars) mice. Data represent mean ± SEM. ***P < 0.001, ****P < 0.0001. UB, upper blade; LB, lower blade; R, rostral; C, caudal.

Exploration of a novel environment is reported to induce Arc expression in the dentate granule cell layer (Guzowski *et al*. 1999; Ramirez-Amaya, 2005; 2013). To test whether repeated exposure to the experimental arena altered the number of Arc^+^ cells, we allowed the mice to freely explore the testing arena once daily for four consecutive days (20 min per session) and subsequently tested in the APA task with the shock zone now being turned on or remaining off (Fig. 2A). The overall distance covered did not change over the four days (repeated measures one-way ANOVA, *F*_1.8,12.7_ = 2.94, *P* = 0.09 for day; day 1: 107 ± 6 m; day 2: 119 ± 6 m; day 3: 123 ± 6 m; day 4: 123 ± 6 m; n = 8). Similarly, mice explored the arena to a similar extent over the four days; the percentage of time spent within the inner zone, which represents 50% of the total arena, also did not change with repeat exposure (repeated measures one-way ANOVA; *F*_2.6,18.3_ = 1.1, *P* = 0.36 for day of testing; day 1: 18.2 ± 1.1%; day 2: 18.3 ± 1.2%; day 3: 16.7 ± 1. %; day 4: 16.4 ± 1%; n = 8). On day 5, mice mastered the APA task efficiently (repeated measures one-way ANOVA *F*_1.9, 5.7_ = 7.2, *P* = 0.027 for intervals; Bonferroni’s *post hoc* analysis: *P* = 0.09 for 0–5 min vs. 15–20 min intervals, n = 4). In this paradigm, familiarization of the mice with their environment is evident from the fact that the 4-day habituated mice received significantly fewer shocks in the 0–5-min interval than those that underwent a single habituation session (compare Fig. 2B with Fig. 1C; unpaired t-test with Welch’s correction, *t* = 5.58, df = 9.99, *P* = 0.0002 for 4-day: 3.25 ± 0.25 shocks (n = 4) vs. single exposure: 5.87 ± 0.40 shocks (n = 8)).

Arc^+^ granule cell numbers in APA and Exp Ctrl mice were significantly increased compared with caged controls (one-way ANOVA: *F*_2, 16_ = 25.64, *P* < 0.0001 for treatment; Tukey’s *post hoc* analysis: *P* = 0.0004 for APA: 23.79 ± 3.04 cells/mm (n = 4) vs. caged: 10.41 ± 1.36 cells/mm (n = 10); *P* < 0.0001 for Exp Ctrl: 26.57 ± 1.69 cells/mm (n = 5) vs. caged: 10.41 ± 1.36 cells/mm (n = 10); Fig. 2D). This effect was recapitulated in all dentate granule cell layer subdomains, with the exception of the lower blade, where Arc expression was not different between APA and Exp Ctrl groups compared with caged controls (repeated measurers two-way ANOVA, *F*_2,16_ = 26.79, *P* < 0.001 for treatment; Bonferroni’s *post hoc* analysis: *P* < 0.0001 for upper blade APA: 32.04 ± 3.21 cells/mm (n = 4) vs. upper blade caged: 12.42 ± 1.85 cells/mm (n = 10); *P* < 0.0001 for upper blade Exp Ctrl: 37.08 ± 2.61 cells/mm (n = 5) vs. upper blade caged: 12.42 ± 1.85 cells/mm (n = 10); Fig. 2E). Arc^+^ granule cells numbers for APA and Exp Ctrl mice in this 5-day testing paradigm were almost identical to those observed in mice that were habituated once only prior to the testing day (unpaired t-test with Welch’s correction, *P* = 0.29 for APA 5-day: 23.11 ± 2.45 cells/mm (n = 5) vs. APA 2-day: 26.49 ± 1.64 cells/mm (n = 8), *t* = 1.15 df = 7.5; *P* = 0.69 for Exp Ctrl 4-day: 26.57 ± 1.69 cells/mm (n = 5) vs. Exp Ctrl 1-day: 25.56 ± 1.70 cells/mm (n = 4), *t* = 0.42 df = 6.85; Fig. 1D compared with Fig. 2D).

### Arc protein expression in the dentate granule cell layer returns to control levels within 12 h of behavioral testing and is upregulated shortly after exposure to a new cage

**Figure 3:**
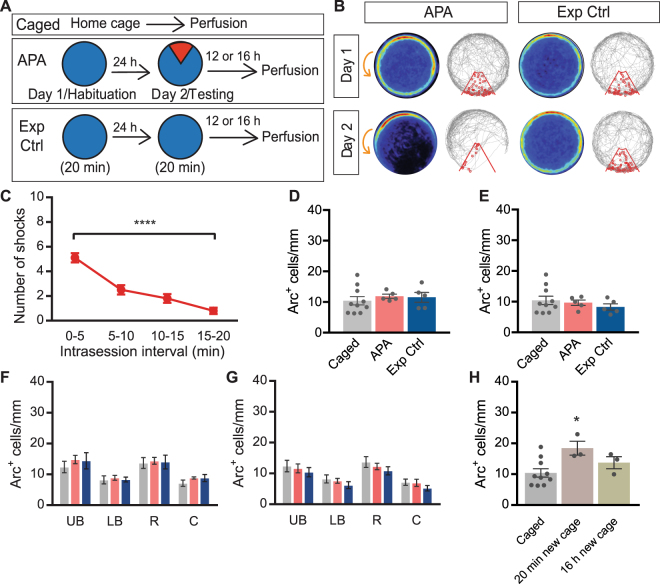
Number of Arc^+^ cells in the granule cell layer returns to baseline levels within 12 h of exposure to experimental arena. **(A)** Schematic representation of the experimental approach. Arena is indicated in blue, while the shock zone is indicated in red. **(B)** Heat maps and representative tracks during Day 1 and Day 2 of APA (left) and Exp Ctrl (exposure control; right) mice, showing APA mice developing place-specific avoidance for the shock zone during the test. Heat maps represent the merged maps of all mice, where the color of a pixel represents the average trajectory of tracks at that location (blue = low, red = high proportion of time). The gray line represents the path of a single mouse and each entry into the shock zone (red sector) is visualized by a small red circle. Orange arrow indicates the counter-clockwise rotation of the testing arena. Performance in the APA task (**C**) and quantification of overall Arc expression in the dentate granule cell layer of APA (red bars) and Exp Ctrl (blue bars) mice 12 h (**D**) and 16 h (**E**) after behavior, compared to caged control mice (gray bars). Quantification of Arc expression in different dentate granule cell layer subdomains of caged mice (gray bars), APA (red bars), and Exp Ctrl (blue bars) mice 12 h (**F**) and 16 h (**G**) after behavior. (**H**) Quantification of Arc expression in the dentate granule cell layer of caged mice (gray bar), 20 min ‘new cage’ mice (light purple bar), and 16 h ‘new cage’ (olive bar) mice. Data represent mean ± SEM. *P < 0.05, ****P < 0.0001. UB, upper blade; LB, lower blade; R, rostral; C, caudal.

As Arc mRNA levels remain elevated in dentate granule cells for at least 8 hours after spatial exploration^15^, and Arc protein is required for both the induction and consolidation of long-term potentiation^1,5–10^, we next explored whether differences in Arc protein expression between experimental groups did emerge at later stages for the condition with the critical and more salient learning, i.e. the APA task. To address this possibility, cohorts of APA and Exp Ctrl mice were tested then perfused either 12 or 16 h later and Arc^+^ cell numbers counted (Fig. 3A). Again, the APA mice quickly learned to avoid the shock zone (repeated measures one-way ANOVA *F*_1.9,17.7_ = 41.07, *P* < 0.0001; Bonferroni’s *post hoc* analysis: *P* < 0.0001 for 0–5 min: 5.10 ± 0.38 vs. 15–20 min: 0.80 ± 0.25; n = 10; Fig. 3B,C). At both 12 h and 16 h after testing, there was no significant difference in the number of Arc^+^ granule cells in either APA and Exp Ctrl mice compared with caged controls (one-way ANOVA: 12 h: *F*_2,17_ = 0.33 *P* = 0.72; caged (n = 10), 12 h APA (n = 5), 12 h Exp Ctrl (n = 5); Fig. 3D; 16 h:: *F*_2,17_ = 0.62 *P* = 0.63; 16 h APA (n = 5), 16 h Exp Ctrl (n = 5); Fig. 3E). There were also no significant region-specific differences in Arc expression between APA or Exp Ctrl mice 12 h and 16 h after testing, compared with caged controls (12 h: repeated measures two-way ANOVA F_2,17_ = 0.30, *P* = 0.74 for treatment, Fig. 3F; 16 h repeated measures two-way ANOVA: F_2,17_ = 0.73, *P* = 0.50 for treatment, Fig. 3G).

To also test the impact of experiencing a more subtle change in the environment on Arc expression, mice were placed into a clean ‘new cage’ and then subsequently perfused either 20 min or 16 h later (Fig. 3H). Twenty minutes after being placed into a new cage, there was a significant increase in the number of Arc^+^ cells within the dentate granule cell layer compared with caged controls (one-way ANOVA: *F*_2,13_ = 4.57, *P* = 0.03; Tukey’s *post hoc* analysis: *P* = 0.027 for 20 min: 18.45 ± 2.26 cells/mm (n = 3) vs. caged: 10.41 ± 1.36 cells/mm (n = 10**))**. Sixteen hours after being placed into a new cage, there was no significant difference in the number Arc^+^ cells within the dentate granule cell layer compared with caged controls (one-way ANOVA: *F*_2,13_ = 4.57, *P* = 0.03; Tukey’s *post hoc* analysis: *P* = 0.46 for 16 h: 9.97 ± 1.95 cells/mm (n = 3) vs. caged: 10.41 ± 1.36 cells/mm (n = 10)).

### Arc expression is not preferentially upregulated in young adult-born granule cells after acquisition of spatial learning

**Figure 4:**
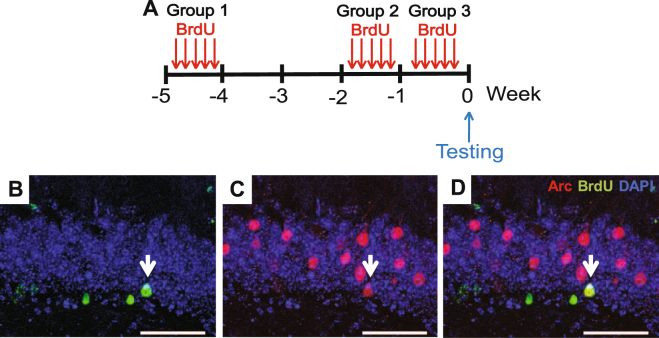
Arc protein expression is not preferentially increased in adult-born, <5-week-old granule cells after acquisition of the APA task. (**A**) Schematic representation of the experimental approach. (**B**–**D**) Representative images of BrdU^+^ cells (green), Arc^+^ cells (red), and a BrdU^+^/Arc^+^ cell (white arrow) in the dentate granule cell layer. All cells are labeled with the nuclear stain DAPI (blue). Scale bars represent 50 µm; n = 4–5.

Having demonstrated that the number of Arc^+^ cells is non-selectively upregulated in the overall granule cell population after acquisition of the APA task, the response of young adult-born dentate granule cells, which are more excitable and exhibit greater synaptic plasticity than mature granule cells^21,24^, was tested. For this experiment, BrdU was administered to mice 0–1, 1–2, or 4–5 weeks before being tested in the APA task (Fig. 4A). There were no significant differences in the number of BrdU^+^/Arc^+^ cells between caged, APA and Exp Ctrl mice at any time point (0–1-week-old cells: one one-way ANOVA: *F*_2,11_ = 1.13, *P* = 0.35, caged 0 ± 0 cells/mm (n = 4), APA: 0.064 ± 0.045 cells/mm (n = 5), Exp Ctrl: 0.057 ± 0.024 cells/mm (n = 5); 1–2-week-old cells: one-way ANOVA: *F*_2,11_ = 0.57, *P* = 0.58, caged 0.02 ± 0.2 cells/mm (n = 4), APA: 0.087 ± 0.046 cells/mm (n = 5), Exp Ctrl: 0.09 ± 0.05 cells/mm (n = 5); 4–5-week-old cells: one-way ANOVA: *F*_2,13_ = 0.65, *P* = 0.53, caged: 0.04 ± 0.03 cells/mm (n = 4), APA: 0.06 ± 0.02 cells/mm (n = 6), Exp Ctrl: 0.08 ± 0.03 cells/mm (n = 6). Additionally, there were no significant differences in the percentage of BrdU^+^ cells expressing Arc between caged, APA and Exp Ctrl mice at any time point (0-1 week cells: one-way ANOVA: *F*_2,8_ = 1.5, *P* = 0.28, caged 0 ± 0 cells/mm (n = 3), APA: 0.37 ± 0.37 cells/mm (n = 4), Exp Ctrl: 0.7 ± 0.23 cells/mm (n = 4); 1–2-week-old cells: one-way ANOVA: *F*_2,11_ = 0.69 *P* = 0.52, caged 0.16 ± 0.16 cells/mm (n = 4), APA: 0.45 ± 0.21 cells/mm (n = 5), Exp Ctrl: 0.61 ± 0.34 cells/mm (n = 5); 4–5-week-old cells: one-way ANOVA: *F*_2,13_ = 0.50, *P* = 0.62, caged 0.33 ± 0.20 cells/mm (n = 4), APA: 0.45 ± 0.15 cells/mm (n = 6), Exp Ctrl: 0.63 ± 0.24 cells/mm (n = 6). These absolute values were minimal, with the 4–5-week-old BrdU-labeled group values representing a total of two BrdU^+^/Arc^+^ cells across all four caged mice, four BrdU^+^/Arc^+^ cells across all five APA mice, and six BrdU^+^/Arc^+^ cells across all five Exp Ctrl mice (Fig. 4B–D). These data indicate Arc is not upregulated in 1-week-old, 1–2-week-old, or 4–5-week-old adult-born granule cells following acquisition of the APA task, and also that these cells do not appear to be preferentially recruited during this task, at least not at the specified ages.

### Number of Arc^+^ cells is reduced in the upper blade of the dentate gyrus in aged mice

**Figure 5:**
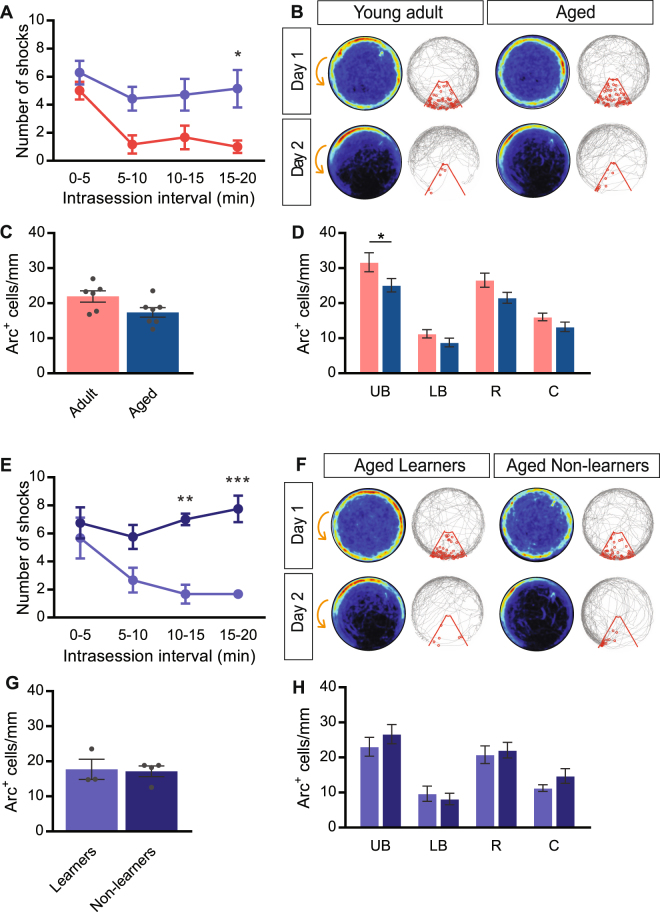
Number of Arc^+^ cells in the granule cell layer (upper blade) is reduced in aged mice. **(A)** Performance of adult 3-month-old mice (red) and ‘aged’ 25-month-old mice (blue) in the APA task. **(B)** Heat maps and representative tracks during Day 1 and Day 2 of testing. Young adult (left) and aged (right) mice develop place-specific avoidance for the shock zone during the test. Heat maps represent the merged maps of all mice, where the color of a pixel represents the average trajectory of tracks at that location (blue = low, red = high proportion of time). The gray line represents the path of a single representative mouse and each entry into the shock zone (red sector) is visualized by a small red circle. Orange arrow indicates the counter-clockwise rotation of the testing arena. **(C)** Quantification of Arc expression in the dentate granule cell layer of adult (red bars) and aged (blue bars) mice. **(D)** Quantification of Arc expression in different dentate granule cell layer subdomains of adult and aged mice. **(E)** Performance of ‘Learner’ (light blue) and ‘Non-learner’ (dark blue) 25-month-old mice. **(F)** Heat maps and representative tracks during habituation (top) and testing (bottom) of ‘Learner’ (left) and ‘Non-learner’ (right) aged mice, showing worse performance of ‘Non-learner’ mice during the test compared with ‘Learner’ mice. **(G)** Quantification of Arc expression in the dentate granule cell layer of ‘Learner’ (light blue bars) and ‘Non-learner’ (dark blue bars) aged mice. **(H)** Quantification of Arc expression in different dentate granule cell layer subdomains of ‘Learner’ (light blue bars) and ‘Non-learner’ (dark blue bars) aged mice. Data represent mean ± SEM. *P < 0.05, **P < 0.01, ***P < 0.001. UB, upper blade; LB, lower blade; R, rostral; C, caudal.

‘Aged’ 25-month-old mice performed significantly worse in the APA task compared with 3-month-old ‘young adult’ mice, as evidenced by the significantly higher number of shocks received at the 15–20-min interval (repeated measures two-way ANOVA: *F*_1,11_ = 7.15, *P* = 0.021 for age, *F*_3,33_ = 10.88, *P* < 0.0001 for interval; Bonferroni’s *post hoc* analysis for 15–20 min interval: *P* = 0.01 for adult 1.0 ± 0.4 shocks (n = 6) vs. aged: 5.1 ± 1.3 shocks (n = 7); Fig. 5A,B). There was a strong trend toward a reduced number of Arc^+^ cells in the dentate granule cell layer of aged mice, compared with young adult mice (unpaired t-test with Welch’s correction *t* = 2.16, df = 10.4, *P* = 0.055, for adult: 21.9 ± 1.6 (n = 6) vs. aged: 17.4 ± 1.4 (n = 7); Fig. 5C). Examination of the subdomains of the dentate granule cell layer revealed significantly less Arc^+^ cells in aged mice compared with young adult mice (repeated measures two-way ANOVA: *F*_1,11_ = 4.45 *P* = 0.059 for age; Bonferroni’s *post hoc* analysis: *P* = 0.03 for upper blade: aged 25.1 ± 1.9 cells/mm (n = 7) vs. adult 31.6 ± 2.7 cells/mm (n = 6); Fig. 5D). Although the aged mice overall performed significantly worse than young adult mice, we observed clear heterogeneity in the aged cohort’s performance, hence aged mice were split into ‘Learners’ and ‘Non-learners’^34,35^. The performance cut-off for aged Learners was a ≥50% decrease in the number of shocks received in the 15–20-min interval compared with the 0–5-min interval of the APA task. Non-learners were defined by a <50% decrease in number of shocks received. A significant difference in APA task performance of Learners and Non-learners was observed at the 10–15-min and 15–20-min intervals (repeated measures two-way ANOVA: *F*_1,5_ = 22.28 *P* = 0.005 for age; Bonferroni’s post hoc analysis: *P* = 0.002 for 10–15-min interval and *P* = 0.0005 for 15–20-min interval; Fig. 5E). The number of Arc^+^ cells within the dentate gyrus was not found to be different between Learners and Non-learners (unpaired t-test with Welch’s correction: *t* = 0.17, df = 3.09, *P* = 0.87, n = 3 Learners and n = 4 Non-Learners; Fig. 5G). In addition, Arc^+^ cell numbers within the dentate subdomains were not different between Learners and Non-learners (repeated measures two-way ANOVA: *F*_1,5_ = 0.36 *P* = 0.57 for age; Fig. 5H). Additionally, there was no correlation between the number of Arc^+^ cells in the dentate granule cell layer and performance of the aged Learners in the APA task (*P* = 0.59, R^2^ = 0.17).

## Discussion

Arc has been widely used as a marker of neurons activated by specific behaviors, particularly those involved in memory formation and recall, with Arc mRNA expression being upregulated in a relatively small subset of neurons in the hippocampus after acquisition of spatial learning in the Morris water maze^3,30^. Few (if any) studies have, however, explored whether differences in Arc protein in this part of the brain can be detected between conditions where a test animal is either allowed to express natural roaming behavior (i.e., free spatial exploration), or where it must learn to not enter into a certain area of the same space through associative learning in order to avoid a negative stimulus. In the present study, we conducted an extensive analysis of Arc^+^ cell numbers in the dentate gyrus – a part of the hippocampus that is thought to play a critical role in learning, memory formation and spatial coding – following exploration of the APA testing arena under conditions where the shock zone remained off or was turned on after habituation.

Learned conditioned avoidance behavior in the APA task relies on both short- and long-term memory formation, with the mice quickly learning to associate the relative position of visual cues in their environment with the location of the shock zone. As a direct result of this learning, the mice constantly and actively adjust their position within the testing arena, receiving less shocks over time in multi-day/multi-trial paradigms^20,36^, and they also progressively enter into the shock zone later in subsequent trials compared to the first trial^36^.

Young adult mice were demonstrated to effectively and reproducibly learn the APA task over a 20-minute timespan, thus providing a suitable paradigm to address the question of whether there are learning-related changes in Arc expression. We found that Arc^+^ granule cell numbers in the dentate gyrus were significantly increased after acquisition of the APA task, but similarly so following spatial exploration of the same testing environment with the shock zone turned off. Consistent with previous reports^15,17^, the greatest number of Arc^+^ granule cells was found in the upper blade of the dentate gyrus. As advanced aging is known to be associated with impaired learning and memory, and Arc mRNA levels are reduced in the hippocampus of aged memory-impaired mice^37^, we also explored how Arc^+^ cell numbers related to the cognitive abilities of aged mice. While an overall (26%) reduction in the number of Arc^+^ cells was detected in the upper blade of aged mice compared to young adult mice, once again, our data did not reveal any obvious correlation between Arc expression and associative learning performance within the aged mouse cohort. Overall, these findings indicate that increases in Arc^+^ cell numbers in the dentate gyrus are not necessarily indicative and/or specific to the acquisition of associative spatial learning; rather, they appear to be more reflective of generalized stimulation in response to an environmental change.

Basal levels of Arc in the hippocampal granule cell layer are normally low, but fluctuations from basal levels are fast detected following application of a stimulus (e.g. novel spatial, tactile or odor cues)^38,39^. Indeed, Arc mRNA expression in the granule cell layer of the dentate gyrus rapidly increases in response to spatial exploration, peaking at approximately 30 min post-exposure but remaining elevated for at least 8 h^7^. In an attempt to minimize the generalized effect of exposure to a new (i.e., testing) environment, we repeatedly placed the mice into the testing arena (up to 4 times) with the shock zone turned off prior to behavioral testing. While mice with such repeat exposure displayed accelerated APA acquisition, receiving significantly fewer shocks in the first interval (0–5 minutes) compared to their counterparts who had only been exposed to the rig once before, the increase in Arc^+^ cell numbers in the dentate gyrus remained the same and was also not different from that observed in mice who were able to explore the testing arena with the shock zone turned off. These findings are consistent with those of Guzowski and colleagues (2006), who reported that repeat exposure to a behavioral arena continues to induce Arc mRNA expression in the hippocampus if done on consecutive days^40^. Arc expression in such a scenario thus appears to be mostly influenced by recent behavioral experiences, irrespective of whether a negative stimulus was present to stimulate associative learning. Burghardt *et al*. (2012) only compared Arc^+^ granule cell numbers between caged and APA mice, but not also free spatial exploration of the testing arena with the shock zone turned off^33^; evidence directly and unequivocally linking Arc expression to associative learning is therefore scant. Similar to the present observations, these authors reported an increase in Arc^+^ cell numbers in APA mice compared to their caged counterparts, and no change in Arc staining after a conflict was created in the associative learning paradigm (changed shock zone position) under homeostatic conditions, i.e. in absence of perturbed neurogenesis; a correlation between the number of Arc^+^ cells and task performance was only observed following brain irradiation.

One potential explanation for the fact that we did not observe a correlation between APA performance and Arc protein expression in young adult mice compared to other studies may be the fact that, in the present study, Arc^+^ neurons were only counted in one particular subregion of the hippocampus, i.e. the granule cell layer of the dentate gyrus; by contrast, Guzowski and colleagues (2001) quantified total Arc mRNA for the entire hippocampus^3^. Whether differences in Arc protein expression exist for other regions of the hippocampus that are known to play a role in spatial learning (e.g., CA1 and CA3)^41^ following acquisition of the APA task thus remains to be determined, although no clear induction of Arc protein over baseline/background was observed in these regions as part of the present study. It should also be recognized that making comparisons on outcomes between different behavioral tasks is somewhat hampered by the fact that they require different levels of hippocampal processing, which is thought to be greater for the APA task than the Morris water maze task^42^, with the former being more similar to pattern separation tasks where the ability of animals to discriminate between minimal changes in the environment is tested^20,43^. Lastly, while acute stress and electrical stimulation can induce expression of IEGs^1,44^, we do not believe this to be a confounding factor as no differences in the number of Arc^+^ cells were observed between mice that received foot shocks during acquisition of the APA task and those that explored with the shock zone turned off, including after repeat exposures. Others have also shown that the mild foot shocks, similar to those delivered in our APA paradigm, do not alter serum levels of the stress hormone corticosterone in mice^36^; hence, any influence of the acute stress response on Arc^+^ cell numbers in the APA task is thus likely negligible.

Finally, while the dentate gyrus is mostly comprised of developmentally generated mature neurons, it also houses a population of young adult-born granule cells that arise from resident precursor cells through the process of adult neurogenesis^45–47^. These newborn neurons (specifically, those that are 4–6 weeks old) exhibit enhanced synaptic plasticity and excitability compared with their older mature counterparts^21,48^ and have been hypothesized to be uniquely, or even preferentially, involved in hippocampal-dependent spatial learning and memory^30,49,50^. Therefore, we predicted that there would be specific and/or preferential Arc protein expression in this subpopulation compared to the overall dentate granule cell population following APA acquisition. Contrary to our expectation, very few (~0.3–0.6%) of the newly generated, 4–5 week-old granule cells displayed somatic Arc protein expression under either control ‘caged’ or behavioral ‘APA’ or ‘Exp Ctrl’ conditions, and with no observed difference in Arc immunostaining levels. The lack of co-localization with Arc is not easily explained by the age/maturation stage of these adult-born neurons as previous work showed that ~7% of 3-week-old, 35% of 4-week old and 75% of 5–11 week-old granule cells express somatic Arc following high-frequency stimulation^51^. The present findings are, however, at odds with previous studies claiming that ~2–4% of these cells expressed Arc as well as other IEG’s like c-Fos and zif268 after acquisition of the Morris water maze task^52,53^. The basis for this discrepancy is not clear, as the only major difference between the approaches used in this and other studies is the behavioral paradigm. We speculate that constant swimming in the Morris water maze may be more stressful than intermittent foot shocks in the APA task, and that this difference may specifically affect Arc expression in young adult-born granule cells^36,54,55^. Consistent with this idea, basolateral amygdala activity has been shown to regulate IEG expression in adult-born granule cells^56^; however, further studies are required to determine whether the Morris water maze is indeed more stressful for mice than the APA task and, if so, whether this is linked to the proportion of young adult-born granule cells that expresses Arc protein.

In conclusion, we suggest that rather than being a reporter of associative spatial learning, the number of Arc^+^ granule cells in the dentate gyrus is more reflective of generalized behavioral stimulation in response to environmental change. The observed re-induction of Arc expression in the dentate gyrus in response to repeated free exploration (i.e. exploration that is not goal-directed) of an already familiar environment would be consistent with the proposed role of the hippocampus in the automatic encoding of new and/or ongoing experiences^57^. It will now be important for future studies to further examine the expression profile of Arc in conjunction with other markers of neuronal activation and plasticity^37^, as it may be that a combination of factors is required to precisely identify those neurons that are important for successful acquisition of the APA task; changes in Arc protein distribution within activated neurons during associative spatial learning should also be assessed. Overall, our findings have important implications for future studies on the role of Arc and other IEGs in spatial learning and memory, highlighting the importance of employing appropriate control conditions that consider as many general features of behavioral testing as possible when trying to investigate molecular mechanisms behind higher cognitive functions such as associative spatial learning and memory.

## References

[1] Lyford GL (1995). Arc, a growth factor and activity-regulated gene encodes a novel cytoskeleton-associated protein that is enriched in neuronal dendrites. Neuron.

[2] Guzowski JF, McNaughton BL, Barnes CA, Worley PF (1999). Environment-specific expression of the immediate-early gene Arc in hippocampal neuronal emsembles. Nat. Neurosci..

[3] Guzowski JF, Setlow B, Wagner EK, McGaugh JL (2001). Experience-dependent gene expression in the rat hippocampus after spatial learning: A comparision of the immediate-early genes, Arc, c-fos, and zif268. J. Neurosci..

[4] Kubik S, Miyashita T, Guzowski JF (2007). Using immediate-early genes to map hippocampal subregional functions. Learn. Mem..

[5] Shepherd JD, Bear MF (2011). New views of Arc, a master regulator of synaptic plasticity. Nat. Neurosci..

[6] Plath N (2006). Arc/Arg3.1 is essential for the consolidation of synaptic plasticity and memories. Neuron.

[7] Ramirez-Amaya V, Angulo-Perkins A, Chawla MK, Barnes CA, Rosi S (2013). Sustained transcription of the immediate early gene Arc in the dentate gyrus after spatial exploration. J. Neurosci..

[8] Shires KL, Aggleton JP (2008). Mapping immediate-early gene activity in the rat after place learning in a water-maze: the importance of matched control conditions. Eur. J. Neurosci..

[9] Denny CA (2014). Hippocampal memory traces are differentially modulated by experience, time, and adult neurogenesis. Neuron.

[10] Beique J, Na Y, Kuhl D, Worley PF, Huganir RL (2010). Arc-dependent synapse-specific homeostatic plasticity. Proc. Natl. Acad. Sci. USA.

[11] Chowdhury S (2006). Arc interacts with the endocytic machinery to regulate AMPA receptor trafficking. Neuron.

[12] Guzowski JF (2000). Inhibition of activity-dependent Arc protein expression in the rat hippocampus impairs the maintenance of long-term potentiation and the consolidation of long-term memory. J. Neurosci..

[13] Kelly MP, Deadwyler SA (2002). Acquisition of a novel behavior induces higher levels of arc mRNA than does overtrained performance. Neuroscience.

[14] Messaoudi E (2007). Sustained Arc/Arg3.1 synthesis controls long-term potentiation consolidation through regulation of local actin polymerization in the dentate gyrus *in vivo*. J. Neurosci..

[15] Ramirez-Amaya V (2005). Spatial exploration-induced Arc mRNA and protein expression: Evidence for selective, network-specific reactivation. J. Neurosci..

[16] Vazdarjanova A, Guzowski JF (2004). Differences in hippocampal neuronal population responses to modifications of an environmental context: Evidence for distinct, yet complementary, functions of CA3 and CA1 ensembles. J. Neurosci..

[17] Chawla MK (2005). Sparse, environmentally selective expression of Arc RNA in the upper blade of the rodent fascia dentata by brief spatial experience. Hippocampus.

[18] Davis CD, Jones FL (2004). & Derrick, B. E. Novel environments enhance the induction and maintenance of long-term potentiation in the dentate gyrus. J. Neurosci..

[19] Xu L, Anwyl R, Rowan MJ (1998). Spatial exploration induces a persistent reversal of long-term potentiation in rat hippocampus. Nature.

[20] Vukovic J (2013). Immature doublecortin-positive hippocampal neurons are important for learning but not for remembering. J. Neurosci..

[21] Ge S, Yang CH, Hsu KS, Ming GL, Song H (2007). A critical period for enhanced synaptic plasticity in newly generated neurons of the adult brain. Neuron.

[22] Toni N (2008). Neurons born in the adult dentate gyrus form functional synapses with target cells. Nat. Neurosci..

[23] Lacefield CO, Itskov V, Reardon T, Hen R, Gordon JA (2012). Effects of adult-generated granule cells on coordinated network activity in the dentate gyrus. Hippocampus.

[24] Marin-Burgin A, Schinder AF (2012). Requirement of adult-born neurons for hippocampus-dependent learning. Behav. Brain Res..

[25] Ikrar T (2013). Adult neurogenesis modifies excitability of the dentate gyrus. Front. Neural Circuits.

[26] Chia C, Otto T (2013). Hippocampal Arc (Arg3.1) expression is induced by memory recall and required for memory reconsolidation in trace fear conditioning. Neurobiol. Learn. Mem..

[27] Czerniawski J (2011). The importance of having Arc: Expression of the immediate-early gene Arc is required for hippocampus-dependent fear conditioning and blocked by NMDA receptor antagonism. J. Neurosci..

[28] Hayes NL, Nowakowski RS (2000). Exploiting the dynamics of S-phase tracers in developing brain: Interkinetic nuclear migration for cells entering versus learning the S-phase. Dev. Neurosci..

[29] Wojtowicz JM, Kee N (2006). BrdU assay for neurogenesis in rodents. Nat. Protoc..

[30] Kee N, Teixeira CM, Wang AH, Frankland PW (2007). Preferential incorporation of adult-generated granule cells into spatial memory networks in the dentate gyrus. Nat. Neurosci..

[31] Gage, G. J., Kipke, D. R. & Shain, W. Whole animal perfusion fixation for rodents. *J. Vis. Exp*. **3565** (2012). 10.3791/3564 PMC3476408 22871843

[32] Franklin, K. B. J. & Paxinos, G. *The mouse brain in stereotaxic coordinates third edition*. Third Edition edn, (Elsevier, 2007).

[33] Burghardt NS, Park EH, Hen R, Fenton AA (2012). Adult-born hippocampal neurons promote cognitive flexibility in mice. Hippocampus.

[34] Frick KM, Baxter MG, Markowska AL, Olton DS, Price DL (1995). Age-related spatial reference and working memory deficits in the water maze. Neurobiol. Aging.

[35] Gage FH, Dunnett SB, Bjorklund A (1984). Spatial learning and motor deficits in aged rats. Neurobiol. Aging.

[36] Lesburgueres E, Sparks FT, O’Reilly KC, Fenton AA (2016). Active place avoidance is no more stressful than unreinforced exploration of a familiar environment. Hippocampus.

[37] Qiu, J. *et al*. Decreased Npas4 and Arc mRNA Levels in the Hippocampus of Aged Memory-Impaired Wild-Type But Not Memory Preserved 11beta-HSD1 Deficient Mice. *J Neuroendocrinol* **28**, doi:10.1111/jne.12339 (2016). 10.1111/jne.12339 PMC4737280 26563879

[38] French PJ (2001). Subfield-specific immediate early gene expression associated with hippocampal long-term potentiation *in vivo*. Eur. J. Neurosci..

[39] Clark PJ, Bhattacharya TK, Miller DS, Rhodes JS (2011). Induction of c-Fos, Zif268, and Arc from acute bouts of voluntary wheel running in new and pre-existing adult mouse hippocampal granule neurons. Neuroscience.

[40] Guzowski JF (2006). Recent behavioral history modifies coupling between cell activity and Arc gene transcription in hippocampal CA1 neurons. Proc Natl Acad Sci USA.

[41] Squire LR (1992). Memory and the hippocampus: a synthesis from findings with rats, monkeys, and humans. Psychol. Rev..

[42] Cimadevilla JM, Wesierska M, Fenton AA, Bures J (2001). Inactivating one hippocampus impairs avoidance of a stable room-defined place during dissociation of arena cues from room cues by rotation of the arena. Proc. Natl. Acad. Sci. USA.

[43] Deng W, Mayford M, Gage FH (2013). Selection of distinct populations of dentate granule cells in response to inputs as a mechanism for pattern separation in mice. Elife.

[44] Cullinan WE, Herman JP, Battaglia DF, Akil H, Watson SJ (1995). Pattern and time course of immediate early gene expression in rat brain following acute stress. Neuroscience.

[45] Stone SS (2011). Functional convergence of developmentally and adult-generated granule cells in dentate gyrus circuits supporting hippocampus-dependent memory. Hippocampus.

[46] Vukovic J, Blackmore DG, Jhaveri D, Bartlett PF (2011). Activation of neural precursors in the adult neurogenic niches. Neurochem. Int..

[47] Nakashiba T (2012). Young dentate granule cells mediate pattern separation, whereas old granule cells facilitate pattern completion. Cell.

[48] Marin-Burgin A, Mongiat LA, Pardi MB, Schinder AF (2012). Unique processing during a period of high excitation/inhibition balance in adult-born neurons. Science.

[49] Kempermann G (2002). Why new neurons? Possible functions for adult hippocampal neurogenesis. J. Neurosci..

[50] Restivo L, Niibori Y, Mercaldo V, Josselyn SA, Frankland PW (2015). Development of Adult-Generated Cell Connectivity with Excitatory and Inhibitory Cell Populations in the Hippocampus. J. Neurosci..

[51] Jungenitz T, Radic T, Jedlicka P, Schwarzacher SW (2014). High-frequency stimulation induces gradual immediate early gene expression in maturing adult-generated hippocampal granule cells. Cereb. Cortex.

[52] Jessberger S, Kempermann G (2003). Adult-born hippocampal neurons mature into activity-dependent responsiveness. Eur. J. Neurosci..

[53] Snyder JS, Radik R, Wojtowicz JM, Cameron HA (2009). Anatomical gradients of adult neurogenesis and activity: Young neurons in the ventral dentate gyrus are activated by water maze training. Hippocampus.

[54] Ons S, Marti O, Armario A (2004). Stress-induced activation of the immediate early gene Arc (activity-regulated cytoskeleton-associated protein) is restricted to telencephalic areas in the rat brain: Relationship to c-fos mRNA. J. Neurochem..

[55] Harrison FE, Hosseini AH, McDonald MP (2009). Endogenous anxiety and stress responses in water maze and Barnes maze spatial memory tasks. Behav. Brain Res..

[56] Kirby ED (2012). Basolateral amygdala regulation of adult hippocampal neurogenesis and fear-related activation of newborn neurons. Mol. Psychiatry.

[57] Morris RG, Frey U (1997). Hippocampal synaptic plasticity: role in spatial learning or the automatic recording of attended experience?. Philos. Trans. R. Soc. Lond. B. Biol. Sci..

